# Actin Remodeling Defects Leading to Autoinflammation and Immune Dysregulation

**DOI:** 10.3389/fimmu.2020.604206

**Published:** 2021-01-07

**Authors:** Riccardo Papa, Federica Penco, Stefano Volpi, Marco Gattorno

**Affiliations:** Center for Autoinflammatory Diseases and Immunodeficiencies, IRCCS Istituto Giannina Gaslini, Genoa, Italy

**Keywords:** pyrin, Wiskott-Aldrich syndrome, autoinflammatory diseases, cytoskeleton, actin

## Abstract

A growing number of monogenic immune-mediated diseases have been related to genes involved in pathways of actin cytoskeleton remodeling. Increasing evidences associate cytoskeleton defects to autoinflammatory diseases and primary immunodeficiencies. We reviewed the pathways of actin cytoskeleton remodeling in order to identify inflammatory and immunological manifestations associated to pathological variants. We list more than twenty monogenic diseases, ranging from pure autoinflammatory conditions as familial Mediterranean fever, mevalonate kinase deficiency and PAPA syndrome, to classic and novel primary immunodeficiencies as Wiskott-Aldrich syndrome and DOCK8 deficiency, characterized by the presence of concomitant inflammatory and autoimmune manifestations, such as vasculitis and cytopenia, to severe and recurrent infections. We classify these disorders according to the role of the mutant gene in actin cytoskeleton remodeling, and in particular as disorders of transcription, elongation, branching and activation of actin. This expanding field of rare immune disorders offers a new perspective to all immunologists to better understand the physiological and pathological role of actin cytoskeleton in cells of innate and adaptive immunity.

*“Cosa bella e mortal passa e non dura.”*

Francesco Petrarca

## Introduction

**Figure 1:**
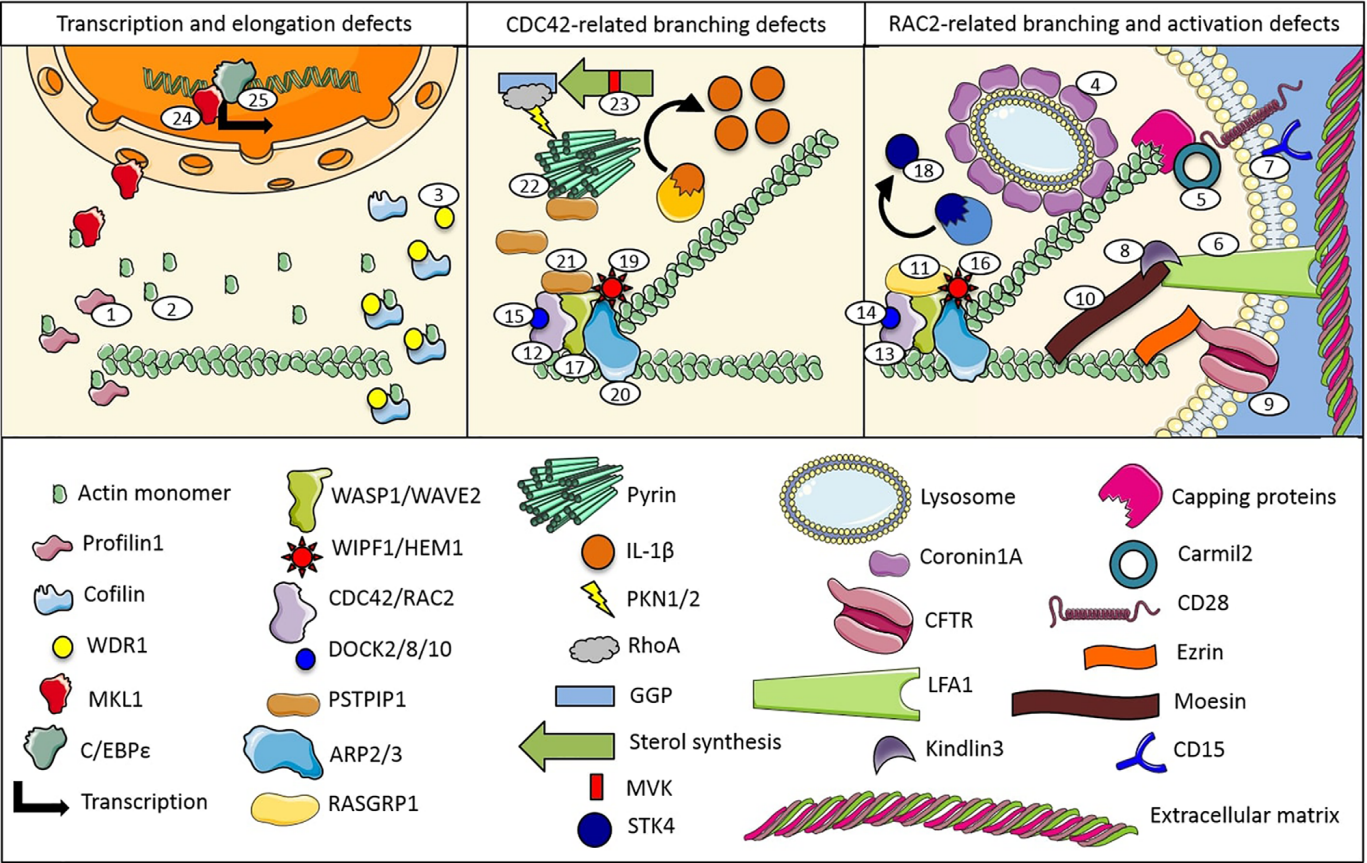
Proteins and pathways involved in monogenic immune system diseases characterized by actin remodeling defects (numbers are related to the manuscript and **Table 1**).

Actin is a family of globular proteins that form microfilaments of cell cytoskeleton. In the past, the most important function of actin was related to the binding of myosin, collaborating to the muscle contraction with troponin. These properties can easily be tested adding pure myosin to water and actin, causing an increase in viscosity and birefringence of the liquid due to the formation of the actomyosin complex (1). Thus, the term of actinopathies was originally considered for a well-defined group of monogenic muscle diseases secondary to the actomyosin complex dysfunction (2). During the recent years, a growing number of disorders of the immune system have been linked to actin cytoskeleton abnormalities (numbers are related to the **Table 1** and **Figure 1**) (3). Furthermore, evidences that actin cytoskeletal deregulation in immune cells causes inflammatory manifestations are increasing (4). In this review, we illustrate the inflammatory and immunological disorders associated with different pathways of actin-binding molecules.

**Table 1:** Monogenic immune system diseases characterized by actin remodeling defects.

N	Location	Gene	Protein	Mechanism	Effect	Diseases	MIM	Inheritance	Main symptoms	Main laboratory characteristics
**Elongation defects**										
1	17p13.2	PFN1	Profilin 1	LOF	Failure to differentiate pre-osteoblast	Early-onset Paget’s disease	None	AR	Polyostotic Paget’s disease, osteosarcome	None
2	7p22.1	ACTB	Beta-actin	GOF	Failure to polarize cytoskeleton in response to fMLP	ACTB-related immunodeficiency	102630	DN	Recurrent stomatitis and otitis media, tuberculosis pneumonia, iritis, keratoconjunctivitis acne, polyarthralgia, intellectual impairment, and short stature	Thrombocytopenia, poor neutrophil chemotaxis and oxidative burst
3	4p16.1	WDR1	WDR1	LOF	Defect of cofilin activation	PFIT	None	AR	Recurrent fevers and stomatitis, microstomia, *Pneumocystis jiroveci* pneumonia, pyoderma gangrenosum, genital ulcers, septic arthritis, and necrotizing cellulitis	Thrombocytopenia, neutrophil and lymphoid dysfunction, hyperferritinaemia
**Activation defects**										
4	16p11.2	CORO1A	Coronin1A	LOF	Defect of WDR1 activation	Coronin1A deficiency	615401	AR	Mycobacterial and viral infections, neurological disorders	Naive T-cells lymphopenia
5	16q22.1	RLTPR	Carmil2	LOF	Defective regulation of capping protein and CD28-mediated costimulation in T-cell	CARMIL2 deficiency	618131	AR	Bacterial and fungal infections, atopy, disseminated EBV-positive smooth muscle tumors	T-cells functional defect
6	21q22.3	ITGB2	ITGAL/M/X	LOF	Deficit of the beta-2 integrin subunit of the LFA-1 causing delayed motility of neutrophils	LAD type I	116920	AR	Recurrent bacterial infections, delayed separation of the umbilical cord, and delayed wound healing	Severe granulocytosis
7	11p11.2	SLC35C1	GDP-L-fucose transporter	LOF	Deficit of CD15 causing delayed motility of neutrophils	LAD type II/CDG2C	266265	AR	LAD1-like immune deficiency, psychomotor retardation, mild dysmorphism	Severe granulocytosis, Bombay blood type
8	11q13.1	FERMT3	Kindlin-3	LOF	Deficit in inside-out signaling that enable high-avidity binding of integrin to ligands on leucocytes and platelets	LAD type III/I variant	612840	AR	LAD1-like immune deficiency, Glanzmann thrombasthenia-like bleeding problems, osteopetrosis	Severe granulocytosis
9	7q31.2	CFTR	CFTR	LOF	Defect of monocyte adhesion	LAD type IV/Cystic fibrosis	219700	AR	Recurrent lung infections, pancreatic insufficiency, male infertility	Hypergammaglobulinemia
10	Xq11	MSN	Moesin	LOF	Impaired T cells proliferation, migration and adhesion	X-MAID	300988	XLR	Recurrent bacterial and varicella zoster virus infections, eczema and other skin manifestations (recurrent molluscum, thrombotic thrombocytopenic purpura), acute stroke	Leukopenia with defective T-cell proliferation and fluctuating neutropenia, hypogammaglobulinemia, ADAMTS13+ thrombocytopenia
**Protrusion defects**										
11	15q14	RASGRP1	RasGRP1	LOF	Defect in Ras activation in T-cells and B-cells	RASGRP1 deficiency	618534	AR	Bacterial and viral infections, autoimmunity	T-cells and B-cells functional defect
12	1p36.12	CDC42	CDC42	GOF	Dysregulation of cytoskeleton	NOCARH/TKS	616737	AD	Fever, rash, lymphedema	Cytopenia, hemophagocitosis, macrothrombocytopenia
13	22q13.1	RAC2	RAC2	LOF/GOF	Defect in fMLF-induced actin remodeling; increased neutrophil superoxide production	RAC2 dysfunction	608203	AR/AD/DN	Recurrent sterile abscesses (frequently perirectal)	Low-normal T and B cells number, hypogammaglobulinemia, leukocytosis with neutrophilia,
14	5q35.1	DOCK2	DOCK2	LOF	Deficit of RAC2 activation	DOCK2 deficiency	616433	AR	Early-onset invasive bacterial and viral infections, autoimmunity	Lymphopenia and defective lumphocytes migration
15	9p24.3	DOCK8	DOCK8	LOF	Deficit of CDC42 activation	DOCK8 deficiency	243700	AR	Recurrent viral infections, early-onset malignancy, and atopic dermatitis	Lymphopenia, hypergammaglobulinemia, mild-to-moderate eosinophilia
16	12q13.13	NCKAP1L	HEM1	LOF	Deficit of WAVE regulatory complex	HEM1 deficiency	None	AR	Recurrent sinopulmonary infections, asthma, hepatosplenomegaly and lymphadenopathy	Increased T and memory T cells, neutrophils migration defects, decreased NK cytotoxicity
**Branching defects**										
17	Xp11.23	WAS	WASP	LOF/GOF	Deficit of ARP2/3 complex activation causing lack of actin branching	WAS/X-linked thrombocytopenia/X-linked neutropenia	301000	XLR	Recurrent bacterial sinopulmonary infections, eczema, autoimmunity, bleeding diathesis	Thrombocytopenia, defective T cell and NK cell functions, increased number of NK cells/Neutropenia
18	20q13.12	STK4	STK4	LOF	Deficit of L-plastin phopshorilation causing abnormal T-cell migration	STK4 deficiency	614868	AR	Recurrent bacterial and viral infections with warts and abscesses, autoimmunity, cardiac malformations	CD4+ and naive CD8+ T-cell and B-cell lymphopenia, neutropenia
19	2q31.1	WIPF1	WIPF1	LOF	Deficit of ARP2/3 complex activation causing lack of actin branching	WAS type 2	614933	AR	WAS-like immune deficiency	Thrombocytopenia, defective T-cell and NK-cell functions, increased number of NK cells
20	7q22.1	ARPC1B	ARPC1B	LOF	Deficit of ARP2/3-dependent F-actin polymerization	PLTEID	617718	AR	Recurrent viral infections, vasculitis, periodic fevers	Thrombocytopenia, hypogammaglobulinemia with high IgE, reduced CD8+ T cell count
21	15q24.3	PSTPIP1	PSTPIP1	GOF	Dysregulation of cytoskeleton resulting in activation of pyrin inflammasome	PAPA, PAMI	604416	AD	Sterile abscesses, pioderma gangrenosum, arthritis	High acute phase reactants
22	16p13.3	MEFV	Pyrin	GOF	Dysregulation of cytoskeleton resulting in activation of pyrin inflammasome	FMF/PAAND	134610	AR/AD	Recurrent fevers with abdominal pain and arthralgia	High acute phase reactants/Neutropenia
23	12q24.11	MKD	Mevalonate kinase	LOF	Dysregulation of cytoskeleton resulting in activation of pyrin inflammasome	MKD	260920	AR	Recurrent fevers, lymphadenopathy, arthralgia, skin rash	High concentration of mevalonate acid in urine during fever attacks
**Transcription defects**										
24	22q13.1	MLK1	MLK1	LOF	Deficit of actin production	MLK1 deficiency	None	AR	Severe bacterial infections (*Pseudomonas* sepsis, malignant otitis media), skin abscesses with abnormal scarring	Mild intermittent thrombocytopenia, selective defect of T-cell proliferation to anti-CD3 antibody, neutrophil phagocytosis and migration defect
25	14q11.2	CEBPE	C/EBPe	LOF/GOF	Deficit in regulation of actin-related genes transcription	SGD/CAIN	245480	AD	Recurrent fevers, skin and tongue abscesses, crater-like oral ulcers, pyoderma gangrenosum, paronychia, enteritis, bleeding diathesis	Atypical Pelger-Huët anomaly with neutrophil hyposegmentation, and impaired chemotaxis, lymph nodal granulomatous inflammation

### Elongation Defects

Actin is the most abundant protein in the majority of eukaryotic cells, contributing to acquire and maintain cell structure and functions. Vertebrates express three actin isoforms, including the α-isoform of skeletal, cardiac, and smooth muscles cells, and the β- and γ-isoforms (5). The conformation of actin monomer, called globular (G)-actin, is the same among different isoforms. G-actin assembles into polarized filaments, called filamentous (F)-actin, that form cortical actin network (CAcN) and cell protusions (6). Monomer binding proteins, such as the Profilin-1, control polymerization. Individual filaments lifetime can be as short as ten seconds or lasting for days, depending on the extracellular stimulus duration and intracellular conditions (7). Inhibiting the actin polymerization through activity of the capping proteins, or stimulating actin disassembly through the Cofilin/actin depolarizing factor (ADF) influences the intracellular concentration of G-actin, usually relatively equal throughout the cell cytoplasm.

Profilin-1 is ubiquitously expressed in human cells (8). Its main function is to chaperone G-actin to the positive-charged barbed end of F-actin in response to an increased concentration of the phosphatidylinositol (4,5)-bi-phosphate (PIP2). Mutation of the *PFN1* gene coding for the Profilin-1 causes the familial form of amyotrophic lateral sclerosis (9) and deletions have been recently related to an early-onset form of Paget’s disease (no. 1 in **Table 1** and **Figure 1**) (10). This condition is characterized by anomalies of the appendicular bone, favoring malign tumors. Pre-osteoblasts lacking Profilin-1 lose their differentiation and adhesion capability and fail to mineralize efficiently the appendicular bone, acquiring invasive properties. Depletion of the Profilin-1 in breast tumor cells causes defects in formation of filopodia, limiting cell motility and favoring proliferation through upregulation of the transcriptional factor SMAD3 (11). On the other hand, deficiency of Profilin-1 acts against invasion of cytotoxic T lymphocytes in tumors and haploinsufficiency of Profilin-1 seems protective against subcutaneous inflammation induced by high fat diet (12). Furthermore, activation of the Profilin-1 pathway has been related to the inflammatory vascular damage in patients with diabetic retinopathy (13, 14).

Heterozygous gain-of-function (GoF) variant of the *ACTB* gene, coding for the β-isoform of actin, has been reported in a female with recurrent infections and defect of neutrophil chemotaxis and oxidative burst (no. 2 in **Table 1** and **Figure 1**) (15). The patient also presented a short stature and intellectual disabilities. No other patients have been reported to date. The authors showed that the mutant β-isoform binds Profilin-1 less efficiently, despite a normal actin polymerization. Loss-of-function (LoF) variants of the *ACTB* gene, as well as of the *ACTG1* gene, coding for the γ-isoform of actin, have been related to the highly variable spectrum of the Baraitser-Winter syndrome, a rare condition without relevant immunological manifestations (16).

Cofilin/ADF activation is dependent by phospholipase Cγ (PLCγ) in tumors and Rac2 signaling in neutrophils (17). Reduction of Cofilin/ADF expression in leukocytes is associated with abnormal chemotaxis (18). In neurons, Cofilin/ADF controls axon elongation and regeneration (19) and serum levels are significantly higher in patient with Alzheimer’s disease (20). Cofilin/ADF is also upregulated in patients with Friedreich’s ataxia, whose mutations correlate with an altered immune-related genes transcription (21, 22).

Proteins containing a short structural motif of approximately 40 amino acids, often terminating in a tryptophan-aspartic acid (WD) dipeptide, called WD40 repeat, can accelerate the Cofilin/ADF activity. The best-known example is the WD40 repeat protein 1 (WDR1), also known as Actin interacting protein 1 (AIP1). Homozygous LoF mutations of the *WDR1* gene cause a monogenic autoinflammatory disease characterized by periodic fever, immunodeficiency, and thrombocytopenia (PFIT; no. 3 in **Table 1** and **Figure 1**) (23, 24). Patients display recurrent fever attacks lasting 3–7 days, every 6–12 weeks, with high acute phase reactants and hyperferritinaemia. Recurrent mucosal inflammation, causing a peculiar acquired microstomia, may resemble the Behcet’s disease’s attacks during childhood (25). Lymphocytes of patients with PFIT show adhesion and motility defects (26). Coronin-1A is another WD40 repeat-containing protein whose LoF mutants have been related to a severe combined immunodeficiency characterized by increased susceptibility to viral and mycobacterial infections (no. 4 in **Table 1** and **Figure 1**) (27–30). Patients usually present with mucocutaneous manifestations, sinopulmonary diseases and neurocognitive disorders without inflammatory manifestations.

On the other hand, the capping proteins are heterodimers composed by two unrelated subunits with highly conserved amino acid sequences. The RGD, leucine-rich repeat, tropomodulin and proline-rich containing protein (RLTPR), also called CARMIL2, is a cytosolic protein that acts as scaffold between the nuclear factor kappa-light-chain-enhancer of activated B cells (NFkB) and CD28 (31, 32). Autosomal recessive (AR) LoF mutations of the *RLTPR* gene cause a primary immunodeficiency (PID) characterized by allergy, increased incidence of bacterial and fungal infections, and virus-related tumors (no. 5 in **Table 1** and **Figure 1**) (33). The abnormal cytoskeleton of T-cell in patients with CARMIL2 deficiency causes defects of activation and is related to an abnormal activity of the capping proteins (34).

### Activation Defects

Over 40 years ago, studies on the ligand-induced movement of immunoglobulin on the surface of lymphocytes called attention to a special relationship between CAcN and antigen-presenting cells (35). A specialized cell–cell junction, the immune synapse (36), is required for the activation of lymphocytes and begin with the formation of thousands of transient, low affinity interactions between antigens and integrins, such as the lymphocyte function-associated antigen 1 (LFA-1) (37). These interactions require a minimum distance of 40 nm, while the major histocompatibility complexes require 15 nm. The consequent antigen-induced CAcN rearrangements leads to morphological changes that are crucial for adhesion, migration, endocytosis, division, gene expression, and calcium flux, as well as for the releasing of cytokines and cytotoxic granules in lymphocytes, neutrophils and monocytes (38).

In particular, on resting leucocytes, LFA-1 is maintained in a low activity state by an inhibitory interaction with the CAcN (39, 40). Therefore, activation of leucocytes requires the release of CAcN-integrin interactions, so that LFA-1 can diffuse in the cell membrane and start binding activities (37). The essential role of CAcN in phagocyte function can be highlighted during chronic infections (41). In fact, microbes are able to lose their integrin ligands in order to escape the immune response (42). The abnormal rolling of leukocytes seems the main affected mechanism in patients with PID caused by LFA-1 defects (nos. 6–9 in **Table 1** and **Figure 1**) (43). The deficiency of the β2 integrin subunit of the LFA-1 causes the leukocyte adhesion deficiency (LAD) type I, and the defective activation of LFA-1 subunits has been related to the LAD type III, both nowadays effectively treated with the hematopoietic stem cells transplantation (44, 45). On the other side, LAD type II is caused by mutations of a fucose transporter gene leading to cell membrane glycans lacking fucosylation. The administration of oral fucose did not seem effective to control the LAD type II clinical manifestations (46, 47).

Finally, a monocyte-selective adhesion defect has been recently noted in patients with cystic fibrosis (CF) and called LAD type IV (48–50). *CFTR* heterozygous LoF variants cause hyper activation of the small G-proteins Rho family that controls integrins activation (51). Interestingly, these small G-proteins are also well-known inhibitor of the pyrin inflammasome (52). Furthermore, CFTR interacts with Ezrin protein *via* its C-terminal domain. Ezrin is the most prominent members of the Ezrin-Radixin-Moesin (ERM) domain-containing protein family that links CAcN to the cell membrane, regulating tension during motility and endocytosis (53, 54). In hematopoietic cells, Ezrin and Moesin are highly expressed, whereas Radixin is mostly absent. Hemizygous LoF mutations of the *MSN *gene coding for Moesin is associated to a PID called X-linked MSN-associated immunodeficiency (X-MAID; no. 10 in **Table 1** and **Figure 1**) (55). Patient T cells displayed impaired proliferative responses after activation by certain mitogens, and a variable defects in cell migration and adhesion, whereas the formation of immunologic synapses is normal. Thus, CAcN dysfunctions impair epithelial tight junction formation as well as lymphocytes adhesion capability in X-MAID patients.

### Protrusions Defects

The collapse of CAcN to the side of cells occupied by microtubule organizing centers creates an opening for new actin polymerization to form membrane protrusions at the leading edge. This process is controlled by the small G-proteins Rho family, including the Cell division control protein 42 homolog (Cdc42) and Rac2 (56).

Small G-proteins are a superfamily of ubiquitously expressed cytosolic hydrolase enzymes that can independently bind and hydrolyze guanosine triphosphate (GTP) to guanosine diphosphate (GDP), becoming inactive (57). The best-known subfamily members are the Ras GTPases that are divided into five main families: Ras, Rho, Ran, Rab, and Arf. The Ras family is generally responsible for cell proliferation, Rho for cell morphology, Ran for nuclear transport and Rab and Arf for vesicle transport. The Ras guanyl nucleotide-releasing protein 1 (RASGRP1) is a diacylglycerol-regulated nucleotide exchange factor specifically activating Ras and regulating T and B cells development, homeostasis and differentiation. Rasgrp1 deregulation in mice results in a systemic lupus erythematosus-like disorder (58) and RASGRP1 deficiency in humans causes a PID characterized by impaired cytoskeletal dynamics (no. 11 in **Table 1** and **Figure 1**) (59). Patients with RASGRP1 deficiency suffer from recurrent bacterial and viral infections especially affecting the lung with a severe failure to thrive and can develop EBV-related lymphomas.

The localization of small G-proteins on the cell membrane is due to their prenylation, a post-translational modification characterized by the addition of twenty-carbon lipophilic isoprene units to the cysteine residues at the C-terminus (60). Furthermore, most of the Rho family members contain a cluster of positively charged residues (i.e., polybasic domain), directly preceding their geranylgeranyl moiety that serves to fine-tune their localization among different cell membrane sites. Overall, the prenylation of small G-proteins is involved in the regulation of cytokines production (61) and can be regulated by statins in monocytes and macrophages (62).

On 2D surfaces, activated Cdc42 and Rac2 generate filopodia and lamellipodia, respectively. The formation of these membrane protrusions consents leucocytes to reach the damaged tissue passing through an intact vessel wall, a process called diapedesis. The local concentration of the complement system C3 fraction also contributes to this process (63). However, in 3D environment, the blebbing motility seems the more common migratory strategy of blood cells (64, 65). Stop-codon variants of the *CDC42* gene has been recently associated with a novel autoinflammatory disease characterized by neonatal-onset of cytopenia, rash, and hemophagocytosis (NOCARH), successfully treated with interleukin-1β inhibition (no. 12 in **Table 1** and **Figure 1**) (66). Furthermore, heterozygous *CDC42* missense variants have been related to the Takenouchi-Kosaki syndrome (TKS) (67–69). TKS patients do not usually display autoinflammatory manifestations but hematologic and/or lymphatic defects, including macrothrombocytopenia, lymphedema, intestinal lymphangiectasia and recurrent infections. Characteristics of platelets and B cells have been recently described (70–72). A recent extensive genotype-phenotype correlation study allows to classify three groups of the *CDC42* variants regarding involved protein domain (73). Based on these evidences, the NOCARH-associated variants occur at the C-terminus that usually allows PIP2 interaction, whereas variants associated with TKS resembling Noonan syndrome occurs at the N-terminus. Thus, different roles of the Cdc42 protein may be subverted in these conditions with different clinical manifestations.

The Rho guanosine triphosphatases Rac2 is expressed only in hematopoietic cells. Patients with Rac2 dysfunction secondary to dominant negative or homozygous LoF mutations present early-onset recurrent abscesses, neutrophilia, and defective wound healing, whereas monoallelic germline GoF mutations of the same *RAC2* gene cause a severe combined immunodeficiency (no. 13 in **Table 1** and **Figure 1**) (74–77). Interestingly, Rac2 activation in neutrophils is primarily mediated by the dedicator of cytokinesis (DOCK) 2, an atypical guanine nucleotide exchange factor (GEF) that rapidly translocate to the plasma membrane in a phosphatidylinositol 3,4,5-trisphosphate (PIP3)-dependent manner upon stimulation, resulting in increased local CAcN polymerization (78, 79). DOCK2 is mainly expressed in peripheral blood leukocytes and DOCK2 deficiency causes an early-onset PID characterized by a T-cell defective chemotactic responses with bacterial and viral infections (no. 14 in **Table 1** and **Figure 1**) (80).

On the other side, DOCK8 is a Cdc42-specific GEF that regulates interstitial migration of dendritic cells and DOCK8 deficiency causes the AR Hyper-IgE syndrome (HIES), a combined immunodeficiency characterized by recurrent viral infections, early-onset malignancy and atopic dermatitis (no. 15 in **Table 1** and **Figure 1**). Patients with DOCK8 deficiency display severe viral skin infections, such as chronic anogenital ulcers, multiple acral warts, and disfiguring molluscum contagiosum (81–84). Selective loss of group 3 innate lymphoid cell has been described in these patients (85).

### Branching Defects

Cdc42 and Rac2 transmit many signals through the GTP-dependent binding of effector proteins containing the Cdc42/Rac interactive binding (CRIB) motif, such as the Wiskott-Aldrich syndrome (WAS) protein (WASP) (86). WASP is restricted to hematopoietic cells, while neuronal WASP (N-WASP), closely related in amino acids sequence, is more widely expressed (87). Other members of this proteins family include the Scar/WAVE proteins. N-WASP has been implicated in filopodia formation downstream of Cdc42, and the Scar/WAVE proteins family has been shown to contribute to the formation of lamellipodia downstream of Rac2. Recently, an immune dysregulation disorders characterized by deficit of the hematopoietic-specific WAVE complex regulator HEM1, coded by the *NCKAP1L* gene, has been characterized (no. 16 in **Table 1** and **Figure 1**) (88). Patients with HEM1 deficiency suffer from recurrent infections, asthma and lymphoproliferation.

N-WASP deficiency increases the production of inflammatory cytokine (89, 90). Human LoF mutations of *WAS* gene cause severe defects in hematopoietic cell functions, leading to the well-known triad of microthrombocytopenia, immunodeficiency, and eczema (no. 17 in **Table 1** and **Figure 1**) (91). The cytoskeletal defects of megakaryocytes are responsible for the low number of platelets in patients with WAS (92). WASP deficiency promotes T-cell cytoskeletal tension decay and phosphorylation of a serine/threonine protein kinase 4 (STK4) that usually increase T-cell migration, therefore promoting immune synapse breaking and secondary B cells dysfunction (93, 94). WASP-deficient lymphocytes fails to differentiate into memory cells (95) and are more prone to develop DNA damages due to the loss of the Golgi-dispersal response, a recently described mechanism of cell survival after ionized radiation exsposure (96). The STK4 deficiency causes a PID characterized by B and T cell lymphopenia, neutropenia, and cardiac malformations (no. 18 in **Table 1** and **Figure 1**) (97). STK4 phosphorylates the Forkhead box O1 transcription factor, increasing NFkB-mediated production of interleukin 12 in dendritic cells and limiting the oxidative stress susceptibility (98). No platelets anomalies have been described in patients with STK4 deficiency. Equally, deficiency of the WASP interacting protein family member 1 (WIPF1) causes a WAS-like syndrome with normal platelet volume (no. 19 in **Table 1** and **Figure 1**). WIPF1 is able to stabilize WASP, preventing its degradation in lymphocytes (99).

ASP controls the rate of actin branching by activating the actin related protein (ARP) 2/3 complex constituted by seven subunits. Two of them, the ARP2 and 3, closely resemble the structure of the G-actin, allowing the formation of a thermodynamically stable dimer that serves as a nucleation site for the new actin filaments at 70° angle from the main filament. Homozygous LoF variants of the *ARPC1B *gene, coding for the p41 regulatory subunits of the ARP2/3 complex, cause the platelet abnormalities with eosinophilia and immune-mediated inflammatory disease (PLTEID; no. 20 in **Table 1** and **Figure 1**) (100–104). Patients with PLTEID usually present systemic inflammation with lymphoproliferation and immunodeficiency resembling WAS, with early onset vasculitis, severe infections, and eczema. A functional test has been recently described to detect asymptomatic carriers (105).

Additional WASP activators include the proline–serine–threonine phosphatase-interacting protein 1 (PSTPIP1), PIP2, and the c-Src protein-tyrosine kinases family. Heterozygous GoF mutation of the *PSTPIP1* gene causes the pyogenic sterile arthritis, pyoderma gangrenosum, and acne (PAPA) syndrome and the PSTPIP1-associated myeloid-related proteinemia inflammatory (PAMI) syndrome (no. 21 in **Table 1** and **Figure 1**) (106, 107). PAMI syndrome is caused by variants that substantially alter electrostatic properties of the PSTPIP1 critical region for auto-inhibiting dimerization, resulting in a GoF mutant protein that constitutively activates the underlying Pyrin inflammasome (108). Pyrin is the pivotal protein of the related inflammasome, a member of cytosolic multiprotein oligomers family responsible for the activation of inflammatory responses in human cells. The Pyrin-associated autoinflammation with neutrophilic dermatosis (PAAND) and familial Mediterranean fever (FMF) are well-known monogenic autoinflammatory diseases both related to GoF variants at different locus sites of the *MEFV* gene and associated with an excessive activation of the Pyrin inflammasome (no. 22 in **Table 1** and **Figure 1**). Recently, the mevalonate kinase deficiency (MKD) caused by homozygous or compound heterozygous LoF mutations in the *MVK* gene has been related to the constitutive activation of Pyrin (no. 23 in **Table 1** and **Figure 1**) (109).

### Production Defects

Megakaryoblastic leukemia 1 (MKL1) is a member of the myocardin-related transcription factors and usually held in an inactive state in the cytoplasm in a reversible complex with G-actin (110). Stimulation of the small Rho GTPases promotes incorporation of G-actin into F-actin, allowing MLK1 to enter into the nucleus, stimulating transcription of actin and other cytoskeletal proteins genes. Homozygous LoF mutation in the *MKL1* gene result in a PID characterized by susceptibility to severe bacterial infection and recurrent skin abscesses (no. 24 in **Table 1** and **Figure 1**) (111). MKL1 deficiency causes reduced phagocytosis and almost complete abrogation of neutrophils spreading properties (112). MLK1 participates in differentiation of megakaryocytes and mild thrombocytopenia has been noted in patients with MKL1 deficiency (113).

Finally, LoF variants of the gene coding for the transcription factor CCAAT enhancer binding protein epsilon (C/EBPϵ) cause a PID called AR neutrophil-specific granule deficiency-1 (SGD) (114), whereas heterozygous GoF variants have been recently related to an autoinflammatory disease called the C/EBPϵ-associated autoinflammation and immune impairment of neutrophils (CAIN; no. 25 in **Table 1** and **Figure 1**). Patients with CAIN display recurrent fevers characterized by abdominal pain, lasting 4–5 days, and skin inflammatory manifestations, such as sterile abscesses, pyoderma gangrenosum and oral ulcerations. The mutant C/EBPϵ causes deregulated transcription of interleukins and interferon response genes in neutrophils (115).

## Discussion

The field of autoinflammation is moving from a gene-centric view of innate immune-mediated diseases towards a systems-based concept, which describes how various convergent molecular pathways, including actin cytoskeleton, contribute to the autoinflammatory process (116) and to a number of conditions characterized by the coexistence of inflammation, autoimmunity and defective immune response. Indeed, the complex regulation of the actin remodeling represents an example of autoinflammatory diseases merging with immunodeficiencies. Despite the wide range of symptoms associated with these disorders, some features may suggest the diagnosis, such as recurrent fevers or infections, atypical skin manifestations (from severe viral infections to eczema and sterile abscesses), cytopenias and defects of chemotaxis and lymphocytes proliferation. Cytopenias may be secondary to the abnormal release of immune cells from the bone marrow and/or impairments in the immune synapsis, while the abnormal diapedesis associated with an altered vessels wall and the increased cell apoptosis in the skin matrix, called cytothripsis, may favor cutaneous manifestations (86). Cytoskeleton-targeted therapies, such as colchicine, may play new roles in these disorders. The study of the molecular and modular diversity of these immune responses to the changing conditions has only recently become possible through the development of the new “omics”-based screening technologies (117). The adoption of “omics” and systems-based concepts will have implications for the discovery of novel diseases and for the possible development of targeted diagnostic tests and treatment options.

## Author Contributions

RP drafted the manuscript. FP, SV, and MG reviewed the manuscript. All authors contributed to the article and approved the submitted version.

## Funding

The study was supported with public funds granted by the Italian Ministry of Health.

## Conflict of Interest

The authors declare that the research was conducted in the absence of any commercial or financial relationships that could be construed as a potential conflict of interest.
